# Rubella and Congenital Rubella Syndrome in the Philippines: A Systematic Review

**DOI:** 10.1155/2016/8158712

**Published:** 2016-12-28

**Authors:** Anna Lena Lopez, Peter Francis N. Raguindin, Maria Asuncion Silvestre, Xenia Cathrine J. Fabay, Ariel B. Vinarao, Ricardo Manalastas

**Affiliations:** ^1^University of the Philippines Manila, National Institutes of Health, Manila, Philippines; ^2^University of the Philippines Manila, Philippine General Hospital, Manila, Philippines; ^3^Kalusugan ng Mag-Ina, Inc (Health of the Mother and Child), Quezon City, Philippines; ^4^Baguio General Hospital and Medical Center, Benguet, Philippines

## Abstract

*Background*. As part of regional elimination efforts, rubella-containing vaccines (RCV) have recently been introduced in the Philippines, yet the true burden of rubella and congenital rubella syndrome (CRS) in the country is largely unknown.* Objective*. To provide baseline information on rubella and CRS prior to routine vaccine introduction in the Philippines.* Methods*. We conducted a systematic literature review on rubella and CRS in the Philippines, including a cross-sectional study conducted in 2002 among 383 pregnant women attending the obstetric outpatient clinic of the Philippine General Hospital to assess rubella susceptibility of women of childbearing age.* Results*. 15 locally published and unpublished studies were reviewed. Susceptibility to rubella among women of childbearing age was higher in rural communities. Retrospective reviews revealed congenital heart diseases, cataracts, and hearing impairments to be most common presentations in children of CRS. In the cross-sectional study, 59 (15.4%) of the 383 pregnant women enrolled were seronegative for rubella IgG.* Conclusion*. Similar to other countries introducing RCV, it was only recently that surveillance for rubella has been established. Previous studies show substantial disabilities due to CRS and a substantial proportion of susceptible women who are at risk for having babies affected with CRS. Establishment of CRS surveillance and enhanced awareness on rubella case detection should be prioritized.

## 1. Introduction

Rubella is a mild viral infection that usually manifests with fever and rash. Its public health significance primarily lies in its ability to cause congenital rubella syndrome (CRS) that results in devastating malformations and significant long-term disability [1–3]. Rubella infection has a teratogenic effect on the developing embryo, which may cause abortion if a woman is infected early in the gestational period or if pregnancy continues, may result in major cardiac anomalies, sensorineural hearing loss, cataracts, or death.

With the use of effective rubella vaccines, rubella was targeted for elimination in two regions of the World Health Organization (WHO) by 2015. But by the end of 2015, only the Region of the Americas was declared to be free of endemic rubella transmission. Worldwide, use of rubella-containing vaccine (RCV) is increasing but despite RCV introduction in 74% of 194 WHO member states, in 2014, global infant immunization coverage remained low at 46% [4]. Surveillance for rubella and CRS is crucial in monitoring the impact of immunization programs to assess disease burden before and after RCV introduction. Although rubella surveillance is being conducted in most countries in conjunction with measles surveillance, surveillance activities for CRS, particularly in developing countries, have proven to be more challenging. Out of 194 member states, only 75 countries began reporting in 2000, which increased to 114 in 2014 but only 14 countries reported positive case identification [4, 5]. Thus, the true burden of CRS remains underestimated [6].

Since 2003, the Western Pacific Region of the World Health Organization (WHO) has resolved to accelerate the control of rubella and prevention of CRS through integration with measles elimination activities [7]. In October 2014, the Western Pacific Region included rubella including CRS elimination as one of eight regional immunization goals specified by the Regional Framework for Implementation of the Global Vaccine Action Plan in the Western Pacific [8].

In the Philippines, RCVs, either singly or in combination with measles and mumps vaccines, had been available in the private sector for more than two decades [9, 10] but this sector is estimated to cover only 5%–10% of the population. RCV was only included in the country's Expanded Programme of Immunization (EPI) in 2010 and given routinely to all infants in 2011 at 12 months of age. Since 2015, through a school-based immunization strategy, children are given a second dose of RCV at school entry (7 years of age) to ensure that children receive at least two doses of measles-containing vaccines. No adult immunization is provided in the public sector and rubella serologic screening of pregnant women is not mandated by the government.

Surveillance for rubella was integrated into the measles surveillance in 2010; however passive laboratory-confirmed surveillance for rubella has been conducted since 2005. In 2009 and 2010, there were 310 and 1,092 serologically confirmed rubella cases out of 1,279 and 4,085 specimens tested, respectively, nationwide [11]. Because there is minimal baseline information on the burden of rubella in the Philippines, we conducted a systematic review of studies on rubella susceptibility, as well as a review on CRS to provide information on rubella and CRS prior to routine vaccine introduction.

## 2. Methods

### 2.1. Systematic Review

For the systematic review, published and unpublished studies on rubella were sought. PubMed searches were performed using the search terms “Philippines” and “rubella” without any limitations on dates. References of the identified publications were reviewed. Literature search in the Philippine databases, namely, HERDIN (Health Research Information and Development Network) and PIMEDICUS (Philippine Index Medicus), was performed using the search terms “rubella,” “CRS,” or “congenital infection.” HERDIN and PIMEDICUS include both locally published and unpublished works. Content experts and specialists were also asked for ongoing and unpublished works on the subject that we may have missed in the local and international database search. Two authors (ALL and PFR) reviewed the retrieved articles and tabulated information in Microsoft Excel™.

### 2.2. Seroprevalence Survey

To determine the proportion of women who were susceptible, that is, seronegative for rubella, a cross-sectional study of pregnant women who consulted in the obstetric outpatient clinic of the Philippine General Hospital (PGH) in the city of Manila for antenatal care was conducted in 2001-2002. Sample size was computed based on the seroprevalence study of Alday [14] that 27% were seronegative in the urban population, and using an interval of 5 with a 95% confidence level, and a 25% dropout rate, then a minimum of 373 pregnant women were required. After obtaining informed consent, data collection forms were completed specifically noting the following: age, occupation, educational attainment, age of gestation, number of previous gestations, and history of rubella immunization. Blood was obtained from the subjects and stored at −20°C until ELISA for rubella was performed. Rubella-specific IgG was tested using commercially available enzyme-linked immunosorbent assay kit (CAPTIA™; Trinity Biotech, Clark Laboratories, TX, USA). ELISA was performed following the manufacturer's instructions.

Data was entered in Microsoft Excel and analysed using Stata 7™ (Stata Corporation, TX, USA). Descriptive statistics for maternal data (age, educational attainment, number of previous gestations, age of gestation, occupation, and history of rubella immunization) and 95% confidence intervals (CI) were calculated. Tests for association of above factors with rubella susceptibility were determined using chi-square test or Fisher's exact test when data were sparse, and statistical significance was set at *p* < 0.05. The study was reviewed and approved by the University of the Philippines Manila-Review Ethics Board prior to patient enrollment. Written informed consent was obtained. Women who had no detectable rubella IgG were informed of the result and advised on the need for immunization.

## 3. Results

### 3.1. Systematic Literature Review

**Figure 1:**
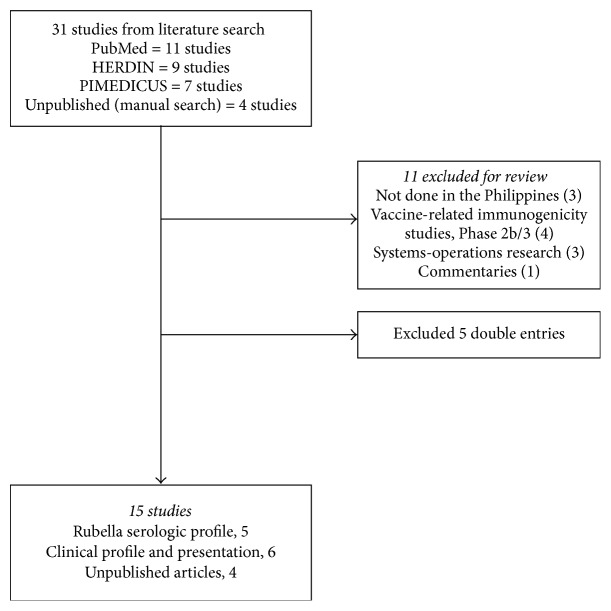
Flow of articles reviewed.

Our search yielded 11 locally published [10, 12–21] and 4 unpublished studies [22–25] (Figure 1), none of which were indexed in PubMed. From 1973–1997, 5 rubella seroprevalence studies were published, 4 of which were conducted in Metro Manila with one including a rural area [10, 12–14], and 1 was in urban Cebu [15] (Table 1). Rubella susceptibility was higher in studies conducted from the 1970s–1980s [10, 12–14] as well as from participants in the rural areas [10, 14].

Ten studies were on CRS [15–24] with one study on pregnancy outcomes [25]. Of the 10 studies on CRS, seven were published (Table 1) and three were unpublished (Table 2). Nine of the 10 studies were retrospective reviews and only one was cross-sectional [18]. Seven of the studies were conducted in a tertiary public hospital (PGH) located in Manila [16, 17, 19, 20, 22–24]. Among published studies, one study proportionately described CRS in congenital cataract cases [19] and two studies described CRS on patients with hearing impairment [18, 21]. Among all patients with congenital cataract, CRS was found to be the most common cause of secondary congenital cataract (28.7% of all congenital cataracts) [19]. CRS was also the most common cause of secondary congenital sensorineural hearing loss [18, 21]. In a prospective study students in a School for the Deaf, 28 (4.8%) children reported other associated impairments (e.g., cataract, blindness, and cardiac defect) suggesting CRS, while 136 (23.5%) reported possible maternal rubella exposure [18].

Only one unpublished study reported on pregnancy outcomes among women who were tested for rubella titers. Out of 124 pregnant women, there were 4 (3.2% of all tested) cases of maternal rubella infection: 1 had fetal death in utero, and 3 babies were diagnosed with CRS [25].

Two studies characterized the clinical profile of CRS among physician-diagnosed cases [17, 20]. The most common clinical manifestations of CRS in the reports were cataract [20] or congenital heart disease [17]. Patent ductus arteriosus and pulmonary stenosis were the most common cardiac manifestations seen in about 45% of the reviewed cases of children with CRS [17].

### 3.2. Cross-Sectional Study

From March 5, 2002, to June 5, 2002, 383 pregnant females participated in the study, of which, 324 (84.6%) had positive rubella IgG by ELISA and 59 (15.4%) were negative or had equivocal results (Table 3). There were no significant differences seen in the rubella immune and susceptible groups as regards age, level of education, place of work, or history of rubella immunization. Only 3 women reported being immunized against rubella in the past, all of whom were seropositive. Among the 12 subjects who were unsure of their rubella immunization, 2 were found to be seronegative. No association was noted between rubella seropositivity and the factors studied (age, educational attainment, more than one pregnancy, age of gestation, and occupation).

## 4. Discussion

Our findings suggest that rubella, including congenital rubella syndrome, is an important public health problem in the Philippines, causing long-term disabilities such as deafness and other congenital disabilities. No prospective studies were conducted in the country on CRS detection, all the studies were retrospective, and most were hospital based; hence most of the cases with CRS that were reported were the ones that required acute care such as congenital heart diseases.

Based on the results of the seroprevalence studies, the number of susceptible women appeared to have declined in the Philippines since the 1980s, wherein 37% of all women tested were susceptible to rubella [14]. In 2002, 15% of pregnant women in an urban setting remained susceptible to rubella. It is likely that the number of susceptible women has not changed substantially as RCV was introduced in the public health setting only in 2010 in young children. It is also likely that rubella susceptibility is higher in rural areas, as was observed in previous rubella susceptibility studies [10, 14].

In 2002, there were 1,666,773 live births in the Philippines [26]; if at least 15% of pregnant women were susceptible to rubella, then a substantial number of pregnancies would have been at risk for CRS. In the Philippines it is estimated that there are 100 to 149 CRS cases per 1,000 live births annually [27].

Similar to developing countries in the Western Pacific Region (WPR), the Philippines has recently introduced RCV. Routine surveillance data for CRS and rubella in the region are just being reported. In Myanmar, the burden of CRS during interepidemic periods was similar to that seen prior to the introduction of RCV in developed countries [28]. Following an outbreak of rubella in Vietnam, surveillance for CRS in Vietnam yielded 292 cases of CRS [29]. In 2014 and 2015, 6 and 7 countries in the WPR have reported >1 confirmed rubella case per 1,000,000 population while no country reported a case of CRS [30]. In the Philippines, reporting CRS cases is not mandatory; hence the disease is likely underestimated. Surveillance on rubella has been integrated in measles-rubella surveillance, wherein patients presenting with rash and fever who tested negative for measles are eventually tested for rubella. This may also be an underestimate as most cases of rubella have an indolent course. Nevertheless, the country reported 3.4 and 1.5 and 1.4 (annualised) laboratory-confirmed rubella cases per 1,000,000 population in 2014, 2015, and 2016, respectively [30].

The WHO recommends two approaches for rubella immunization. The first recommends immunization of adolescent and adult women of childbearing age, aimed at reducing the burden of CRS. The second, which is suitable for elimination of both rubella and CRS, interrupts the transmission of rubella virus by the introduction of RCV into the routine childhood immunization schedule in combination with the vaccination of older age groups [3]. In view of the regional goal of rubella elimination, RCVs have been introduced in the Philippines at 9 and 12–15 months of age as well as at 7 years of age in schools. However, rubella susceptibility screening is not routinely conducted among pregnant women and immunization of women of child bearing age has not been included in the public health setting. Continued vigilance and enhanced surveillance for CRS and rubella are important in identifying cases to attain the regional goal of rubella elimination.

## 5. Conclusions

Our review presents the baseline information on rubella and CRS and supports the establishment of CRS surveillance in the country to identify the burden, as well as for programmatic implementation to monitor the impact of immunization.

## Figures and Tables

**Table:** (a) Serologic surveys (*n* = 5).

Author	Date	Setting	Methods	Inclusion criteria	Confirmatory test	Results
Espiritu-Campos et al. 1973 [12]	1973	Community setting (urban)	Prospective	Male and female volunteers (0–40 years old)	Hemagglutination-Inhibition test	32.2% rubella susceptible

Del Mundo 1973 [10]	1973	Community setting (urban and rural)	Prospective	Male and female volunteers (0–40 years old)	Hemagglutination-Inhibition test	26% rubella susceptible

Chan et al. 1979 [13]	1978	Community setting (urban)	Prospective	Nonpregnant women (6–45 years old)	Hemagglutination-Inhibition test	29.6% were rubella susceptible (38.1% for women ≤ 30 years old, 12.5% for >30 years old)

Alday et al. 1982 [14]	1982	Tertiary hospitals (urban Manila City and rural Las Pinas)	Prospective	Nonpregnant women aged (16–45 years old)	Hemagglutination-Inhibition test	37.3% were rubella susceptible (urban women ≤ 30 years old, 80%; urban women > 30 years old, 20%; and rural women ≤ 30 years old, 64%, rural women > 30 years old, 36%)

Yu et al. 1997 [15]	1997	Tertiary hospital (Cebu City)	Prospective	Pregnant women (17–45 years old)	Microparticle Enzyme Immunoassay (IgG)	10.9% were rubella susceptible

**Table:** (b) Congenital rubella syndrome (*n* = 6).

Author	Month/year study was conducted	CRS manifestation(s) primarily used to identify cases	Methods	Included subjects	Diagnostic criteria for CRS	Results
Nueva-Espana et al. 1988 [16]	January 1981 to June 1986	Hearing loss	Retrospective review of audiology clinic records and audiologic test	Children 0–16 years old presenting in a tertiary hospital with hearing loss (mean 4.7 years old)	Hearing loss plus maternal history of febrile rash and other signs associated with CRS	17 cases (out of 496) had maternal history of rubella, and only 3 cases of the 17 were diagnosed with CRS

Santos-Cabaero et al. 1998 [17]	January 1990 to December 1994	Cardiac, hearing loss, and cataract	Retrospective review of patient records	Children diagnosed with CRS by physicians	Rubella IgM confirmed CRS cases	Deafness and cataract were the most predominant symptoms with 26 (62%) and 28 (67%) cases, respectively. Other clinical findings included were psychomotor delay (50%), congenital heart disease (45%), neonatal cholestasis (31%), and glaucoma (4 cases or 10%)

Yu and Rameriz 2003 [18]	September to October 2002	Hearing loss	Prospective, cross-sectional study using questionnaire	Students enrolled in a School for the Deaf (mean 14.2 years old)	Hearing loss plus maternal history of rubella	136 (23.5%) deaf students had maternal history of rubella, among which 15 (2.6%) had visual problems and 8 (1.4%) had congenital heart disease

Tecson and Santiago 2004 [19]	January 2000 to August 2003	Cataract	Retrospective review of patient records	Infants 0–12 mos with atraumatic cataract in a tertiary hospital	Congenital cataract with history of maternal measles and heart disease	45 cases (out of 218 cases, or 20.5%) had CRS. 18 cases (out of 218 cases or 8.2%) had suspected CRS CRS was the most common cause of secondary cataract

Agnas 2005 [20]	January 1995 to December 2002	Cardiac, cataract, and hearing loss	Retrospective review of patient records	Children diagnosed with CRS by physicians	Rubella IgM confirmed CRS cases	Cataract was the most common clinical manifestation, followed by patent ductus arteriosus at 24 (49%) and 15 cases (31%), respectively. The other clinical manifestations were hepatomegaly (10%), jaundice (10%), pulmonary artery stenosis (6%), extrauterine growth retardation (4%), glaucoma (2%), and hemolytic anemia (2%)

Tipayno 2008 [21]	January 1996 to December 2005	Hearing loss	Retrospective review of audiologic records	Patients tested in audiologic clinic of a pediatric specialty tertiary hospital (mean 3.9 years old)	Clinically diagnosed CRS (criteria used for diagnosis not mentioned)	48 patients out of 2,783 (1.7%) physician-diagnosed CRS 44 patients with CRS (91%) with hearing loss, 80% of which were severe to profound hearing loss

**Table 2:** Unpublished studies (*n* = 4) on congenital rubella syndrome.

Author	Year	Sign	Methods	Included subjects	Results
Tanglao-Salazar 1993 [22]	1993	Hearing loss	Retrospective chart review	Children with hearing impairment	18.4% with maternal history of rubella infection during pregnancy

Rodriguez 1995 [23]	1995	Hearing loss	Retrospective chart review	Children with hearing impairment	13% diagnosed with congenital rubella

Santos-Gonzales and Santiago 2013 [24]	2013	Cataract	Retrospective review of records in pediatric ophthalmology clinic	Children with suspected, probable, or laboratory-confirmed CRS^a^	Out of 23 cases, there were 6 (26%) rubella IgM confirmed, 11 (48%) probable, and 6 (26%) CRS suspected cases. Cataract was seen in 21 (91%) of the cases of CRS. Two patients presented with pigmentary retinopathy

Limgenco 2000 [25]	2000	Pregnancy outcome	Retrospective review	Pregnant women with TORCH^b^ titers	4 cases of maternal rubella infection, 1 had fetal death in utero, and 3 babies were diagnosed with congenital rubella syndrome

^a^The 1997 US CDC criteria for CRS include the following. *Clinical description*: an illness is usually manifesting in infancy resulting from rubella infection in utero and characterized by signs or symptoms from the following categories: (A) cataracts/congenital glaucoma, congenital heart disease (most commonly patent ductus arteriosus, or peripheral pulmonary artery stenosis), loss of hearing, and pigmentary retinopathy; * *(B) purpura, splenomegaly, jaundice, microcephaly, mental retardation, meningoencephalitis, and radiolucent bone disease. *Case classification*:* suspected*: a case with some compatible clinical findings but not meeting the criteria for a probable case; *probable*: a case that is not laboratory confirmed and that has any two complications listed in paragraph (A) of the clinical description or one complication from paragraph (A) and one from paragraph (B) and lacks evidence of any other etiology; *confirmed*: a clinically compatible case that is laboratory confirmed; *infection only*: a case that demonstrates laboratory evidence of infection, but without any clinical symptoms or signs. Laboratory confirmation is through viral isolation, rubella IgM, and rubella antibody level that persists at a higher level and for a longer period than expected from passive transfer of maternal antibody.

^b^TORCH: toxoplasma, rubella, cytomegalovirus, herpes titers.

**Table 3:** Characteristics of pregnant women and their rubella serologic status, 2002.

Variable	*N*	Immune *n* (%, 95% CI^a^)	Susceptible *n* (%, 95% CI^a^)	*P* value
*Age*				0.33
16–25	176	152 (86.36, 80.39–91.06)	24 (13.64, 8.94–19.61)	
26–35	168	142 (84.52, 78.15–89.63)	26 (15.48, 10.37–21.85)	
36–45	39	30 (76.92, 60.67–88.87)	9 (23.08, 11.13–39.33)	
*Education*				0.88
Elementary	21	17 (80.95, 58.9–94.55)	4 (19.05, 5.45–41.91)	
High school	180	152 (84.44, 78.31–89.41)	28 (15.56, 10.59–21.69)	
College	182	155 (85.16, 79.15–89.99)	27 (14.84, 10.01–20.85)	
*Work*				0.21
Primarily at home	294	245 (83.33, 78.57–87.41)	49 (16.67, 12.59–21.43)	
Primarily outside	89	79 (88.76, 80.30–94.47)	10 (11.24, 5.52–19.69)	
*History of rubella immunization*				0.75
None	368	311 (84.51, 80.40–88.05)	57 (15.49, 11.95–19.6)	
Yes	3	3 (100)	0	
Unknown	12	10 (83.33, 51.58–97.91)	2 (16.67, 2.09–48.41)	

^a^Exact binomial 95% confidence interval.
